# Dermatan-4-*O*-Sulfotransferase-1 Contributes to the Undifferentiated State of Mouse Embryonic Stem Cells

**DOI:** 10.3389/fcell.2021.733964

**Published:** 2021-09-23

**Authors:** Chika Ogura, Shoko Nishihara

**Affiliations:** ^1^Department of Bioinformatics, Graduate School of Engineering, Soka University, Hachioji, Japan; ^2^Glycan and Life System Integration Center (GaLSIC), Soka University, Hachioji, Japan

**Keywords:** mouse embryonic stem cells, *D4ST1*, self-renewal, *Cdx2*, endodermal differentiation

## Abstract

Mouse embryonic stem cells (mESCs) have the properties of self-renewal and pluripotency. Various signals and growth factors maintain their undifferentiated state and also regulate their differentiation. Glycosaminoglycans are present on the cell surface and in the cell matrix as proteoglycans. Previously, we and other groups reported that the glycosaminoglycan heparan sulfate contributes to both maintenance of undifferentiated state and regulation of mESC differentiation. It has been shown that chondroitin sulfate is needed for pluripotency and differentiation of mESCs, while keratan sulfate is a known marker of human ESCs or induced pluripotent stem cells. We also found that DS promotes neuronal differentiation from mESCs and human neural stem cells; however, the function of DS in the maintenance of mESCs has not yet been revealed. Here, we investigated the role of DS in mESCs by knockdown (KD) or overexpression (O/E) of the *dermatan-4-O-sulfotransferase-1* (*D4ST1*) gene. We found that the activity of the ESC self-renewal marker alkaline phosphatase was reduced in *D4ST1* KD mESCs, but, in contrast, increased in *D4ST1* O/E mESCs. *D4ST1* KD promoted endodermal differentiation, as indicated by an increase in *Cdx2* expression. Conversely, *Cdx2* expression was decreased by *D4ST1* O/E. Wnt signaling, which is also involved in endodermal differentiation, was activated by *D4ST1* KD and suppressed by *D4ST1* O/E. Collectively, these results demonstrate that D4ST1 contributes to the undifferentiated state of mESCs. Our findings provide new insights into the function of DS in mESCs.

## Introduction

Mouse embryonic stem cells (mESCs) are established from the inner cell mass at the blastocyst stage (Evans and Kaufman, 1981; Martin, 1981). They have the properties of self-renewal and pluripotency, which means that they are capable of differentiation into the three primary germ layers, endoderm, mesoderm, and ectoderm, via the epiblast and primitive endoderm. There are many studies showing that various signals and growth factors contribute to maintenance of undifferentiated state and regulation of differentiation in mESCs. Because the role of glycans in these processes has not been fully elucidated, we previously performed an RNA interference (RNAi) screen to identify glycosyltransferases essential for self-renewal and pluripotency in mESCs. To date, we have identified four glycan structures that are required to maintain the naïve pluripotent state: (1) LacdiNAc structure (GalNAcβ1-4GlcNAc) (Sasaki et al., 2011), (2) heparan sulfate (HS) (Sasaki et al., 2008, 2009; Hirano et al., 2012, 2013), (3) *O*-GlcNAc (Miura and Nishihara, 2016; Miura et al., 2018; Pecori et al., 2021), and (4) T antigen (Galβ1-3GalNAc) (Pecori et al., 2020).

Glycosaminoglycans (GAGs) such as HS are present on the cell surface and in the cell matrix as proteoglycans, consisting of GAG and a core protein. GAGs show diverse structures due to sulfation and have a characteristic disaccharide repeating structure. In addition to HS, keratan sulfate (KS), and chondroitin sulfate (CS)/dermatan sulfate (DS) are well-known GAGs. HS and CS/DS bind to the Ser residue of core proteins through a common linkage region, namely GlcAβ1-3Galβ1-3Galβ1-4Xylβ-*O*-ser (Sugahara and Kitagawa, 2000), while KS binds to core proteins via an *N*-linked or *O*-linked oligosaccharide (Funderburgh, 2002). Previously, we and other groups showed that HS contributes to maintenance of undifferentiated state and regulation of differentiation in mESCs by promoting Wnt, BMP, FGF, and Fas signaling (Johnson et al., 2007; Sasaki et al., 2008, 2009; Kraushaar et al., 2010, 2012; Lanner et al., 2010; Fico et al., 2012; Hirano et al., 2012, 2013). In addition, Izumikawa et al. (2014) reported that CS is required for pluripotency and differentiation of mESCs, while KS is known as a marker of human ESCs or induced pluripotent stem cells (Andrews et al., 1984; Pera et al., 1988; Adewumi et al., 2007; Kawabe et al., 2013). To our knowledge, however, the function of DS in mESCs has not been revealed yet.

In the synthesis of DS, epimerization from glucuronic acid (GlcA) to iduronic acid (IdoA) is initially carried out by dermatan sulfate epimerase (Maccarana et al., 2006) or dermatan sulfate epimerase-like (Pacheco et al., 2009) after synthesis of the CS chain (i.e., GlcA-GalNAc repeating disaccharide structure). Subsequently, dermatan-4-*O-*sulfotransferase-1 (D4ST1) (Evers et al., 2001) transfers sulfate to the C-4 hydroxyl group of GalNAc. Lastly, sulfate is transferred to the C-6-hydroxyl group of GalNAc and the C-2 hydroxyl group of IdoA by *N*-acetylgalactosamine-4-sulfate 6-*O*-sulfotransferase (GalNAc4S-6ST) (Ito and Habuchi, 2000) and uronyl-2-sulfotransferase (UST) (Kobayashi et al., 1999), respectively. While the GalNAc4S-6ST and UST sulfotransferases are common to both CS and DS, D4ST1 is specific to DS.

We previously reported that DS promotes neuronal differentiation from mESCs and human neural stem cells (Ogura et al., 2020). It is also known that D4ST1 is needed for neuronal differentiation from mouse neural stem cells (Bian et al., 2011). Moreover, *D4ST1* deficiency is the cause of Ehlers-Danlos syndrome (EDS), a genetic connective tissue disorder with defects in skin, ligaments, articulation, internal organs, and blood vessels (Kosho, 2016; Malfait et al., 2017).

Here, therefore, we investigated the role of DS in the undifferentiated state of mESCs by knockdown or overexpression of *D4ST1*. We found that D4ST1 contributes to self-renewal of mESCs and *D4ST1* knockdown induces endodermal differentiation by activating Wnt signaling. Our results provide new insights into function of DS in mESCs.

## Materials and Methods

### Cell Culture

The R1 mESC line (Nagy et al., 1993) was cultured on mouse embryonic fibroblasts (MEFs) in mESC culture medium [DMEM (Gibco), 15% FBS (Nichirei Bioscience, Inc.), 1% penicillin/streptomycin (Gibco), 0.1 mM 2-mercaptoethanol (Gibco), 1 mM non-essential amino acids (Gibco), and 1,000 units/ml of LIF (Oriental Yeast)]. MEFs were isolated from embryos at E14.5 and inactivated by the addition of 10 μg/ml of mitomycin C (Sigma).

### Transfection

For transient knockdown (KD) of *D4ST1* in mESCs, we generated siRNA expression vectors using pSilencer 3.1-H1 (Ambion). The siRNA sequences used for RNAi were designed as described previously (Ui-Tei et al., 2004) by using siDirect^1^ :

E*gfp*, 5′-GATCCCGCCACAACGTCTATATCATGGGGAAA ATCCATGATATAGACGTTGTGGCTTTTTTGGAAA-3′; *D4S T1* KD1, 5′-GATCCCCAGCACTACTTCAAGTTCCTGTTTGG CTTCCTGTCACCAAACAGGAACTTGAAGTAGTGCTGTTT TTTA-3′; *D4ST1* KD2, 5′-GATCCCTCCTCTTGCTAGGTCTGA ATCATTTGCTTCCTGTCACAAATGATTCAGACCTAGCAAG AGGATTTTTTA-3′; *D4ST1* KD3, 5′-GATCCCCTTCAAGAT GTGCTACCTAAGGCTTCCTGTCACCTTAGGTAGCACATCT TGAAGTTTTTTA-3′. *Egfp* was used as a negative control.

We also generated a *D4ST1* overexpression (O/E) vector using pCAGI-Puro (a kind gift of Professor Kumiko Ui-Tei). The vector was produced by using the pGEM^®^ -T Easy Vector Systems (Promega) as described previously (Kamiyama et al., 2006). We used an empty vector as a control for the O/E experiments.

Before transfection, we replated the mESCs at 1 × 10^6^ cells on gelatin-coated 60-mm culture dishes (NIPPON Genetics) containing LIF. After 16 h, the cells were transfected with 4 μg of siRNA expression vectors targeting *D4ST1* (*D4ST1* KD1 and *D4ST1* KD2) or *Egfp*, or the *D4ST1* O/E vector by using Lipofectamine 2000 (Invitrogen). At 1 day after transfection (TF day 1), transfected cells were selected by adding 2 μg/ml of puromycin (Sigma). We harvested the cells at TF day 2 for the *D4ST1* O/E experiments or TF day 4 for the *D4ST1* KD experiments.

### Cell Proliferation Assay

*D4ST1* KD mESCs at TF day 4 were replated at 8 × 10^3^ cells per well on gelatin-coated 96-well plates (IWAKI) containing LIF. After 24 h, we counted the number of viable cells by using microscopy.

### Alkaline Phosphatase Staining

The transfected mESCs were replated at 1.25 × 10^5^ cells per well on gelatin-coated 24-well plates (NIPPON Genetics) containing LIF. After 5 days, we carried out ALP staining with a StemTAG^TM^ Alkaline Phosphatase Staining Kit (Cell Biolabs, Inc.). ALP-positive colonies were counted by using microscopy.

### Real-Time PCR

Total RNA was extracted from cells by using TRI Reagent^®^ (Molecular Research Center, Inc.) and reverse-transcribed by using SuperScript^TM^ VILO^TM^ Master Mix (Invitrogen). Real-time PCR was performed by using Quant Studio 12K Flex (Applied Biosystems). The relative amount of each mRNA was normalized against the amount of β*-actin* mRNA in the same sample. The primer sets for real-time PCR are listed in Supplementary Table 1.

### Western Blotting Analysis

The transfected mESCs were lysed with lysis buffer (50 mM Tris–HCl pH 7.4, 150 mM NaCl, 1% Triton X-100, 5 mM EDTA, 1 mM Na_3_VO_4_, 10 mM NaF, and protease inhibitors). The protein samples (5–10 μg) were separated by 8% SDS-PAGE and transferred to PVDF membranes (Millipore). After blocking with 1% BSA/TBST, the membranes were incubated with primary antibodies. The membranes were then incubated with secondary antibodies and Amersham ECL Prime Western Blotting Detection Reagent (GE Healthcare Life Science) was used for detection. The antibodies are listed in Supplementary Table 2.

### Statistical Analysis

Data were compared with unpaired two-tailed Student’s *t*-test or Dunnett test. Asterisks denote statistical significance (n.s., *p* > 0.05; ^∗^*p* < 0.05; ^∗∗^*p* < 0.01; and ^∗∗∗^*p* < 0.001).

## Results

### *D4ST1* Contributes to Self-Renewal of mESCs

**FIGURE 1:**
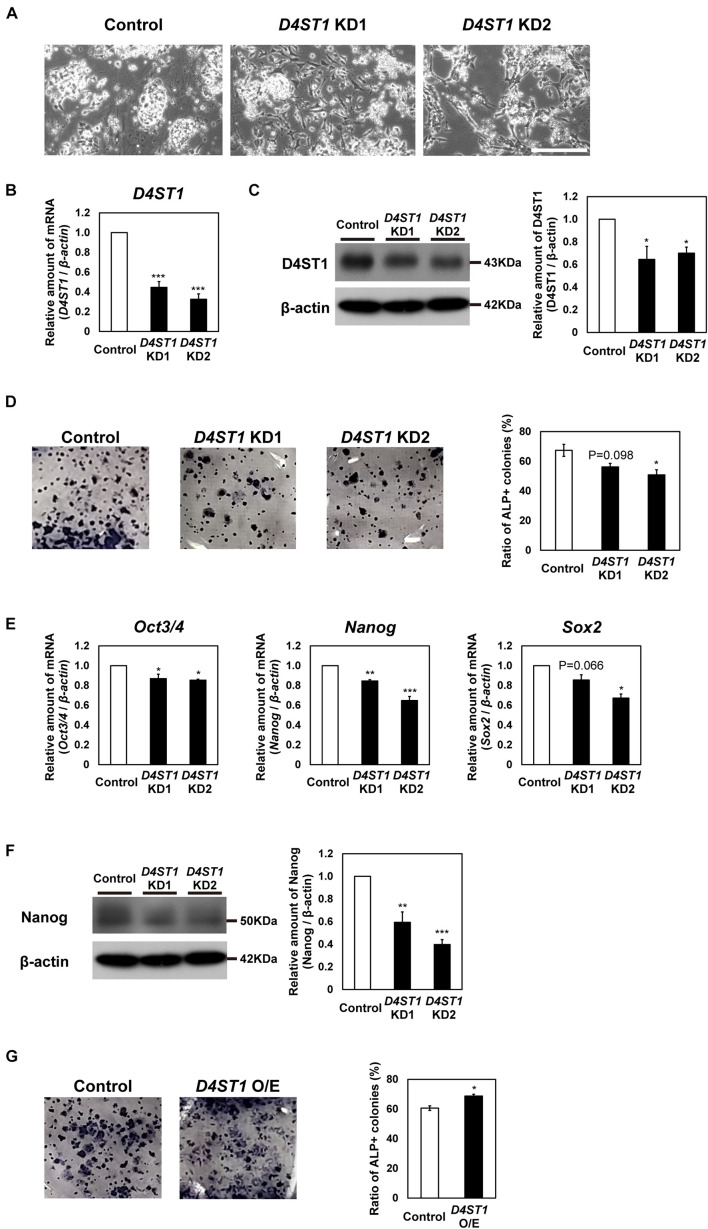
*D4ST1* contributes to self-renewal of mESCs. **(A)** Morphological observation of *D4ST1* KD mESCs at day 4 after transfection (TF day 4). Scale bar: 200 μm. **(B)** Real-time PCR analysis of *D4ST1* in *D4ST1* KD mESCs at TF day 4. The amount of *D4ST1* mRNA was normalized to that of β*-actin* mRNA and is shown relative to the control (set to 1). **(C)** Western blotting analysis of D4ST1 in *D4ST1* KD mESCs at TF day 4. Histogram shows the mean densitometric readings of bands, which were normalized to β-actin and are shown relative to the control (set to 1). **(D)** Alkaline phosphatase (ALP) staining of *D4ST1* KD mESCs at TF day 4. (Left) Representative images of ALP staining. (Right) Histogram showing the ratio of ALP-positive colonies. **(E)** Real-time PCR analysis of pluripotent markers in *D4ST1* KD mESCs at TF day 4. The amounts of pluripotent marker mRNAs (*Oct3/4*, *Nanog*, and *Sox2*) were normalized to that of β*-actin* mRNA and are shown relative to the control (set to 1). **(F)** Western blotting analysis of Nanog in *D4ST1* KD mESCs at TF day 4. Histogram shows the mean densitometric readings of bands, which were normalized to β-actin and are shown relative to the control (set to 1). The representative bands of the loading control (β-actin) are the same as those in **(C)** because the same samples were used for these analyses. **(G)** ALP staining of *D4ST1* O/E mESCs at TF day 2. (Left) Representative images of ALP staining. (Right) Histogram showing the ratio of ALP-positive colonies. The values shown are means ± SD (*N* = 3). Those significantly different to the control by Dunnett test **(B–F)** or unpaired two-tailed Student’s *t*-test **(G)** are indicated as follows: ***p < 0.001; **p < 0.01; *p < 0.05.

To investigate function of DS in mESCs, we performed knockdown (KD) of *D4ST1*, which is the first sulfotransferase in the DS synthesis pathway (Figure 1A). We designed two constructs (*D4ST1* KD1 and *D4ST1* KD2), which expressed different siRNAs targeting *D4ST1* mRNA, and one construct targeting *Egfp* as a negative control. After transfection of mESCs with these constructs, the decreased expression of *D4ST1* mRNA and D4ST1 was confirmed by real-time PCR and western blotting, respectively (Figures 1B,C). Proliferation in *D4ST1* KD mESCs was not changed as compared with control cells (Supplementary Figure 1). To determine self-renewal potential, ALP staining was performed for the *D4ST1* KD1 and KD2 transfected mESCs. The number of ALP-positive colonies was reduced by *D4ST1* KD, indicating that D4ST1 contributes to self-renewal of mESCs (Figure 1D). Furthermore, the expression of three pluripotent markers, *Oct3/4*, *Nanog* and *Sox2*, were decreased in *D4ST1* KD mESCs (Figure 1E and Supplementary Figure 2). The expression of *Klf2* was decreased, while that of *Klf4* and *Rex1* did not change significantly (Supplementary Figure 3). The amount of Nanog was also significantly decreased in *D4ST1* KD mESCs (Figure 1F).

We also examined the effect of overexpression (O/E) of *D4ST1* in mESCs (Supplementary Figure 4A). The increased expression of *D4ST1* mRNA and D4ST1 after transfection with the O/E vector was confirmed by real-time PCR and western blotting, respectively (Supplementary Figures 4B,C). In contrast to *D4ST1* KD, *D4ST1* O/E increased the number of ALP-positive colonies (Figure 1G), confirming that D4ST1 contributes to self-renewal of mESCs.

### Endodermal Differentiation of mESCs Is Induced by *D4ST1* KD

**FIGURE 2:**
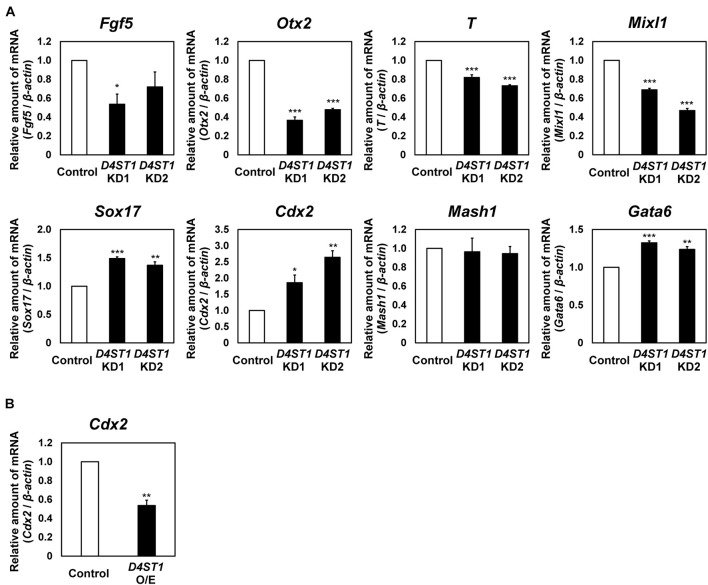
Endodermal differentiation of mESCs is induced by *D4ST1* KD. **(A)** Real-time PCR analysis of differentiation markers in *D4ST1* KD at TF day 4. The amounts of differentiation marker mRNAs (*Fgf5*, *Otx2*, *T*, *Mixl1*, *Sox17*, *Cdx2*, *Mash1*, and *Gata6*) were normalized to that of β*-actin* mRNA and are shown relative to the control (set to 1). **(B)** Real-time PCR analysis of *Cdx2* in *D4ST1* O/E at TF day 2. The amount of *Cdx2* mRNA was normalized to that of β*-actin* mRNA and is shown relative to the control (set to 1). The values shown are means ± SD (*N* = 3). Those significantly different to the control by Dunnett test **(A)** or unpaired two-tailed Student’s *t*-test **(B)** are indicated as follows: ***p < 0.001; **p < 0.01; *p < 0.05.

Next, we examined the expression of differentiation markers to determine which lineages are induced from mESCs by *D4ST1* KD (Figure 2A and Supplementary Figure 2). In *D4ST1* KD mESCs, the expression of two epiblast markers, *Fgf5* and *Otx2*, was decreased. The expression of two mesoderm markers, *T* and *Mixl*, was also significantly decreased. In contrast to mesodermal markers, the expression of two endoderm markers, *Sox17* and *Cdx2*, was significantly increased in *D4ST1* KD mESCs, indicating that the endodermal differentiation was induced in *D4ST1* KD mESCs. Expression of the primitive endoderm marker *Gata6* was significantly increased, indicating that the differentiation to primitive endoderm was also induced in *D4ST1* KD mESCs. However, expression of the ectoderm marker *Mash1* was not changed by *D4ST1* KD. Collectively, these results suggest that D4ST1 contributes to the pluripotency of mESCs.

We also analyzed the expression of *Cdx2*, a marker of hindgut (Beck et al., 1995; Sherwood et al., 2007), in *D4ST1* O/E mESCs. The expression of *Cdx2* was significantly decreased in *D4ST1* O/E mESCs (Figure 2B). It has been reported that *Cdx2* expression is required for differentiation of hindgut (Stringer et al., 2008). Thus, the significantly increased or decreased expression of *Cdx2* in the respective *D4ST1* KD or *D4ST1* O/E mESCs indicates that D4ST1 might regulate endodermal differentiation, including differentiation to hindgut.

### BMP Signaling Is Suppressed and Wnt Signaling Is Activated by *D4ST1* KD

**FIGURE 3:**
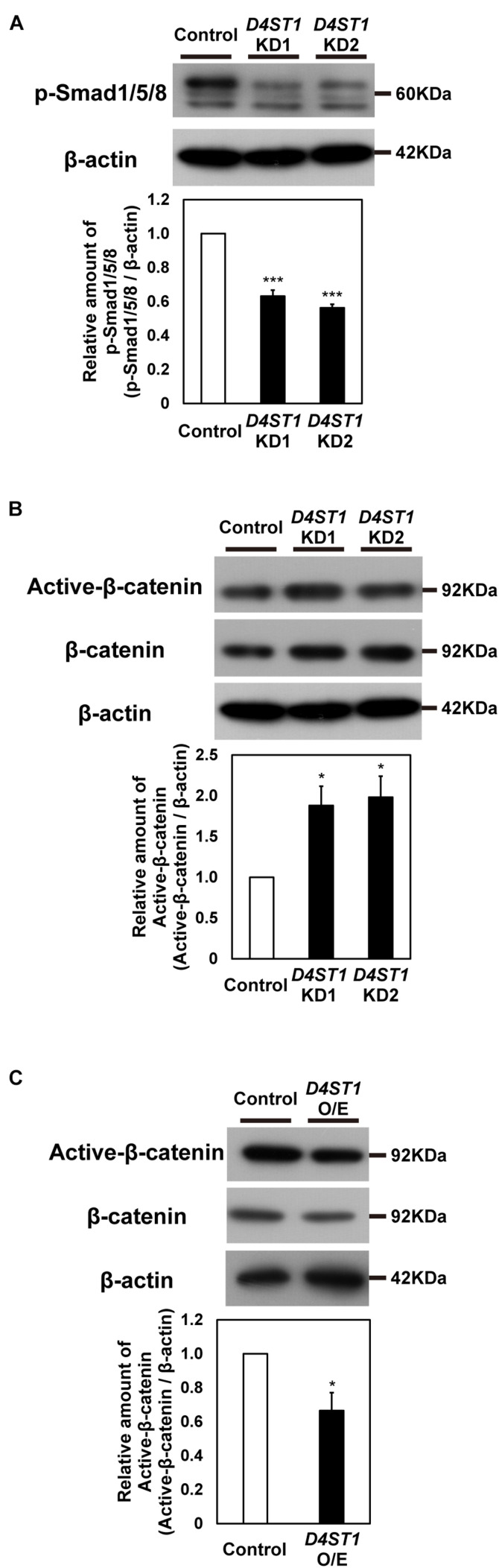
Wnt signaling is activated by *D4ST1* KD. **(A,B)** Western blotting analysis of p-Smad1/5/8 and Active-β-catenin in *D4ST1* KD mESCs at TF day 4. Histogram shows the mean densitometric readings of bands normalized to β-actin, and was shown relative to the control (set to 1). The representative bands of the loading control (β-actin) are the same as those in Figure 1C because the same samples were used for these analyses. **(C)** Western blotting analysis of Active-β-catenin in *D4ST1* O/E mESCs at TF day 2. Histogram shows the mean densitometric readings of bands, which were normalized to β-catenin and are shown relative to the control (set to 1). The values shown are means ± SD (*N* = 3). Those significantly different to the control by Dunnett test **(A,B)** or unpaired two-tailed Student’s *t*-test **(C)** are indicated as follows: ***p < 0.001; *p < 0.05.

To analyze effect of *D4ST1* KD on signaling pathways, we used western blotting to analyze several signaling components in *D4ST1* KD mESCs. First, we examined the BMP/Smad1/5/8 signal, which contributes to self-renewal in mESCs by suppressing neural determination (Ying et al., 2003) and by up-regulating ERK-specific dual-specificity phosphatase 9 to reduce extracellular signal-regulated kinase activity, which is required for cell fate commitment (Li et al., 2012). Phosphorylated Smad1/5/8 was significantly decreased by *D4ST1* KD (Figure 3A). Thus, the reduced activity of the ESC self-renewal marker ALP (Figure 1D) is caused by a decrease in BMP signal.

Second, we examined the Wnt/β-catenin signal which induces endodermal differentiation (Zhong et al., 2017) and subsequently the hindgut domain during primitive gut tube formation in mouse (Engert et al., 2013). Whereas the relative amount of Active-β-catenin was significantly decreased by *D4ST1* O/E (Figure 3C), it was significantly increased (Figure 3B) by *D4ST1* KD. We also analyzed the expression of Wnt signaling target genes in *D4ST1* O/E mESCs; *Lef1* was significantly decreased, while that of *Axin2* and *Cdx1* tended to be decreased (Supplementary Figure 5). These results demonstrate that *D4ST1* KD induces endodermal differentiation and subsequent regionalization of the hindgut domain by activating Wnt signaling.

## Discussion

In this study, we found that self-renewal and the undifferentiated state of mESCs were compromised by *D4ST1* KD. In *D4ST1* KD mESCs, self-renewal of mESCs was reduced and endodermal differentiation was induced. In particular, the expression of *Cdx2*, which is a hindgut marker, was significantly increased by *D4ST1* KD and significantly decreased by *D4ST1* O/E. Similarly, Wnt signal was activated by *D4ST1* KD and suppressed by *D4ST1* O/E.

**FIGURE 4:**
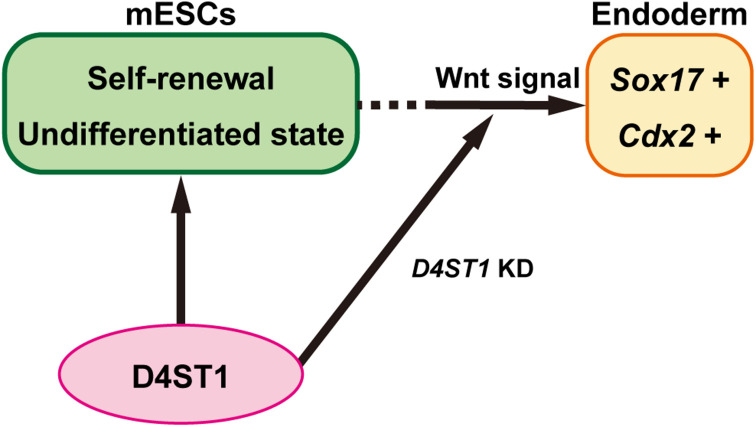
*D4ST1* contributes to the undifferentiated state of mESCs. *D4ST1* contributes to self-renewal and the undifferentiated state of mESCs. Knockdown of *D4ST1* causes promotion of endodermal differentiation by activation of Wnt signaling.

It has been reported that *Cdx2* is essential for determination of intestinal mesoderm or endoderm differentiation (Stringer et al., 2008). The endoderm and mesoderm arise from a transient common precursor cell population referred to as “mesendoderm.” The specification of endoderm requires Wnt/β-catenin signaling, which maintains the expression of Nodal, which in turn promotes the expression of a network of transcription factors within the endodermal lineage including Sox17 (Zorn and Wells, 2009). After endodermal linage determination, the gut tube is formed. The gut tube then becomes regionalized along the dorsal-ventral and anterior-posterior axes into broad foregut, midgut, and hindgut domains (Zorn and Wells, 2009). The Wnt/β-catenin signal also specifies the hindgut domain by inducing *Cdx2* expression, which is required for both the hindgut and positioning of the foregut-hindgut boundary in mouse development (Sherwood et al., 2011). Therefore, our results demonstrate that D4ST1 contributes to the undifferentiated state of mESCs, and *D4ST1* KD induces endodermal and subsequent hindgut differentiation by activating Wnt signaling (Figure 4).

*CDX2* is also required for intestinal development in human pluripotent stem cells, and WNT signaling similarly promotes endoderm-hindgut differentiation (Kumar et al., 2019). Although further investigation is needed to elucidate the function of D4ST1 in human development, *D4ST1* deficiency is known to be one of the causes of EDS (Kosho, 2016). *D4ST1*-deficient EDS presents characteristic craniofacial features, multiple congenital contractures, and progressive joint and skin laxity. Of note, joints and dermis are tissues derived from mesoderm. In the present study, in contrast to endodermal markers, mesodermal markers were suppressed by *D4ST1* KD. Thus, D4ST1 may contribute to mesodermal differentiation in humans. In addition, it has been reported that skin complaints are caused by disorganization of collagen networks due to decorin, a dermatan sulfate proteoglycan (DSPG) (Hirose et al., 2018). There are several DSPGs, including decorin, biglycan, and fibromodulin. Determination of the DSPG that contributes to differentiation of mESCs will be an interesting issue for future study.

Because D4ST1 is a sulfotransferase involved in DS synthesis, it is possible that DS regulates Wnt signaling. GAGs such as HS and CS play a key role in signal transduction as co-receptors or as trappers by binding signal ligands (Wang et al., 2017). For example, DS has been reported to interact with bFGF, FGF7, and EGF (Taylor et al., 2005; Bian et al., 2011). To our knowledge, however, binding of DS to Wnt has not been demonstrated. This is also an interesting issue for future analysis.

In conclusion, we have shown that D4ST1 is required for self-renewal and the undifferentiated state in mESCs and *D4ST* KD induces endoderm differentiation and subsequent hindgut differentiation by activating Wnt signals. This study provides new insights into function of DS in mESCs.

## Data Availability Statement

The original contributions presented in the study are included in the article/Supplementary Material, further inquiries can be directed to the corresponding author.

## Author Contributions

CO and SN: conceptualization. CO: methodology, validation, formal analysis, investigation, data curation, writing – original draft, and visualization. SN: resources, writing – review and editing, supervision, project administration, and funding acquisition. Both authors contributed to the article and approved the submitted version.

## Conflict of Interest

The authors declare that the research was conducted in the absence of any commercial or financial relationships that could be construed as a potential conflict of interest.

## Publisher’s Note

All claims expressed in this article are solely those of the authors and do not necessarily represent those of their affiliated organizations, or those of the publisher, the editors and the reviewers. Any product that may be evaluated in this article, or claim that may be made by its manufacturer, is not guaranteed or endorsed by the publisher.

## Footnotes

1
http://sidirect2.rnai.jp/
