# Supplementary material for: Dermatan-4-O-Sulfotransferase-1 Contributes to the Undifferentiated State of Mouse Embryonic Stem Cells
Source: Front Cell Dev Biol. 2021 Sep 23;9:733964. doi: 10.3389/fcell.2021.733964 (PMC8495257; doi:10.3389/fcell.2021.733964)
Supplement: Supplementary file 1 [file Data_Sheet_1.docx]

Supplementary Material

**Supplemental Table 1. Primers for real-time PCR.**

| *Gene* | Forward (5'-3') | Reverse (5'-3') |
| --- | --- | --- |
| *m.β-actin* | GCTCTGGCTCCTAGCACCAT | GCCACCGATCCACACAGAGT |
| *m.D4ST1* | GCCTGCTCTAACTGGAAACG | CTGCCAGAAACACCAAGTCA |
| *m.Oct3/4* | CTCACCCTGGGCGTTCTCT | AGGCCTCGAAGCGACAGA |
| *m.Nanog* | CAGAAAAACCAGTGGTTGAAGACTAG | GCAATGGATGCTGGGATACTC |
| *m.Sox2* | GTGGAAACTTTTGTCCGAGACC | CGCAGCCGCTTGGC |
| *m.Fgf5* | GCAGCCCACGGGTCAA | CGGTTGCTCGGACTGCTT |
| *m.Otx2* | CATGATGTCTTATCTAAAGCAACCG | GTCGAGCTGTGCCCTAGTA |
| *m.T* | TGCTGCAGTCCCATGATAACTG | ATGACTCACAGGCAGCATGCT |
| *m.Mixl1* | GCACGTCGTTCAGCTCGGAGCAGC | AGTCATGCTGGGATCCGGAACGTGG |
| *m.Sox17* | GCACAACGCAGAGCTAAGCA | CTGCCAAGGTCAACGCCT |
| *m.Cdx2* | GAGCTGGCTGCCACACTTG | GCTTCTTCTTGATTTTCCTCTCCTT |
| *m.Mash1* | CCTCCCCAAACCCCACAT | ACAAAGATTGGAAGCATTCTATGAAG |
| *m.Gata6* | CCCCTCATCAAGCCACAGAA | GTGACAGTTGGCACAGGACAGT |
| *m.Axin2* | GGGAGCAGTTTTGTGGCAGCA | AGGGTCCTGGGTAAATGGGTGAG |
| *m.Lef1* | ACTGTCAGGCGACACTTCCATG | GTGCTCCTGTTTGACCTGAGGT |
| *m.Tcf3* | CCTCTCATCACCTACAGCAACG | CTGGAGACAGTGGGTAATACGG |
| *m.Cdx1* | CAAGGCGGACGCCCTACGAAT | TAGGCGTTGGTGGTCTGTGTAG |

**Supplemental Table 2. Antibodies for western blotting analysis.**

| Antibody | Company | Dilution |
| --- | --- | --- |
| β-actin | Sigma | 1:10000 |
| CHST14 | Thermo Fisher Scientific | 1:1000 |
| Active-β-catenin | Cell Signaling | 1:2000 |
| β-catenin | Cell Signaling | 1:2000 |
| p-Smad1/5/8 | Cell Signaling | 1:2000 |
| Nanog | SANTA CRUZ | 1:1000 |
| mouse IgG HRP | Cell Signaling | 1:10000 |
| rabbit IgG HRP | Cell Signaling | 1:10000 |

**Supplemental Figure 1.**


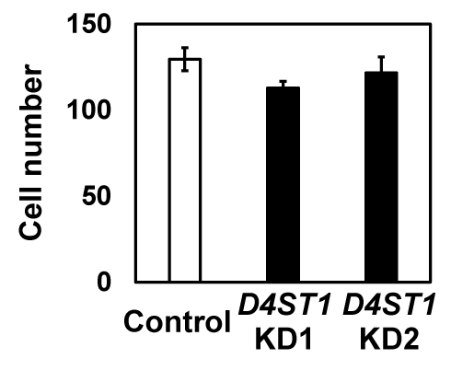


**Supplemental Figure 1. Proliferation did not change in *D4ST1* KD mESCs.**

Shown is a cell proliferation assay in *D4ST1* KD mESCs. The values shown are means ±SD (*N*=3). No significant difference was found by Dunnett test.

**Supplemental Figure 2.**


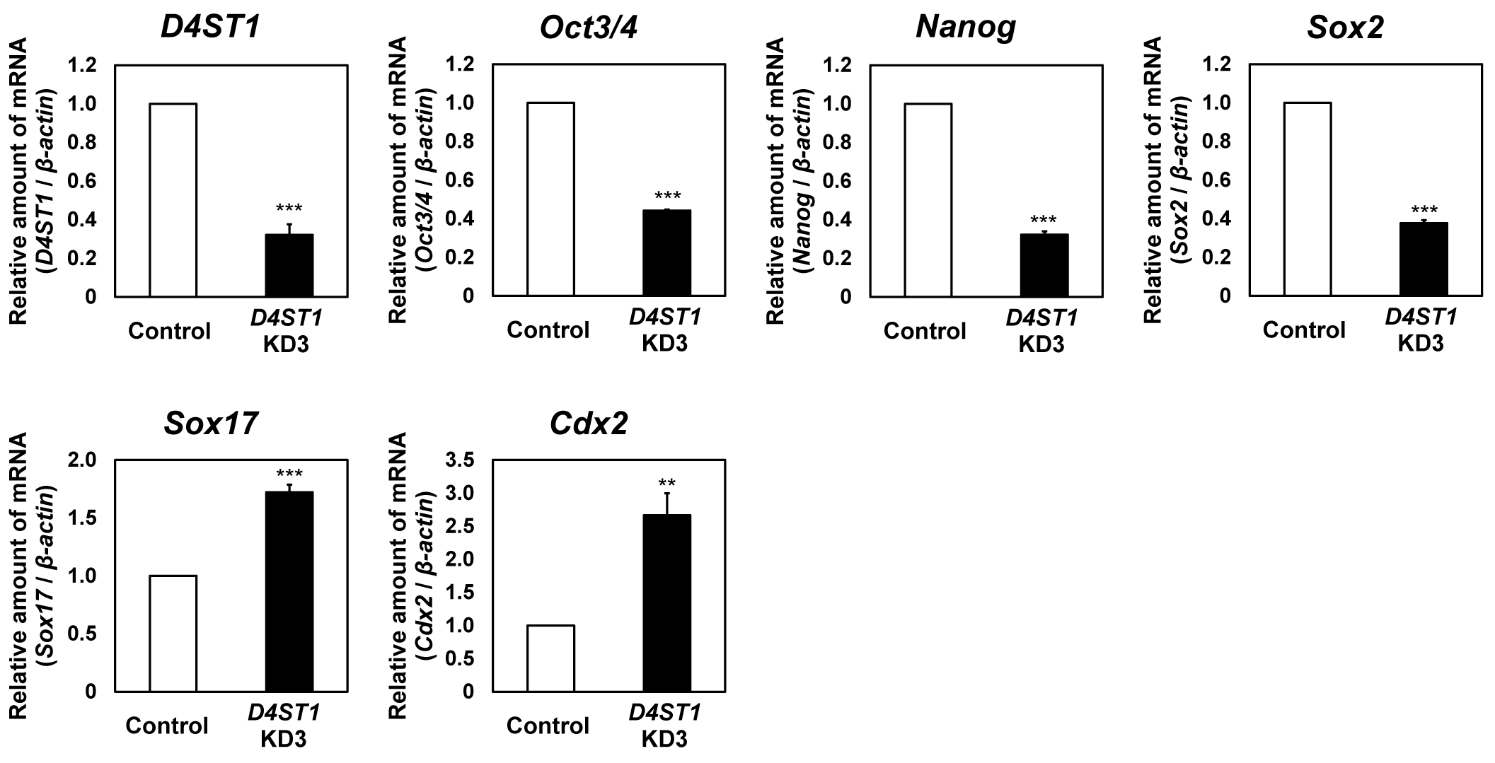


**Supplemental Figure 2. *D4ST1* KD in mESCs caused a decrease in pluripotent marker expression and an increase in endoderm marker expression.**

Shown is real-time PCR analysis of *D4ST1*, pluripotent markers, and endoderm markers in *D4ST1* KD mESCs at TF day4. The mRNA levels of *D4ST1*, pluripotent markers (*Oct3/4*, *Nanog*, and *Sox2*), and endoderm markers (*Sox17* and *Cdx2*) were normalized to that of *β-actin* mRNA and are shown relative to the control (set to 1).

The values shown are means ±SD (*N*=3). Those significantly different to the control by unpaired two-tailed Student’s t-test are indicated as follows: ***, *p*<0.001; **, *p*<0.01.

**Supplemental Figure 3.**


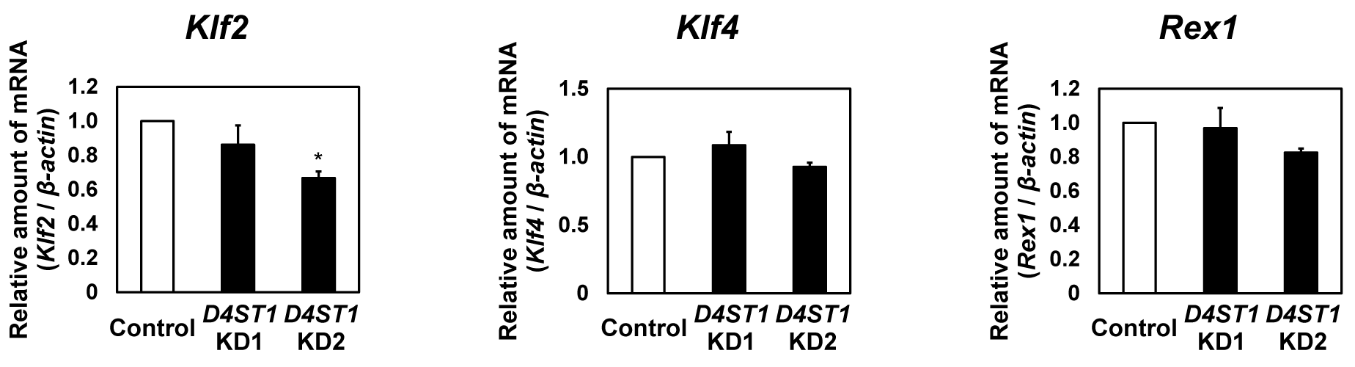


**Supplemental Figure 3. The expression of pluripotency markers in *D4ST1* KD mESCs.**

Shown is real-time PCR analysis of pluripotent markers in *D4ST1* KD mESCs at TF day4. The mRNA levels of pluripotent markers (*Klf2*, *Klf4*, and *Rex1*) were normalized to that of *β-actin* mRNA and are shown relative to the control (set to 1).

The values shown are means ±SD (*N*=3). Those significantly different to the control by Dunnett test are indicated as follows: *, *p*<0.05.

**Supplemental Figure 4.**


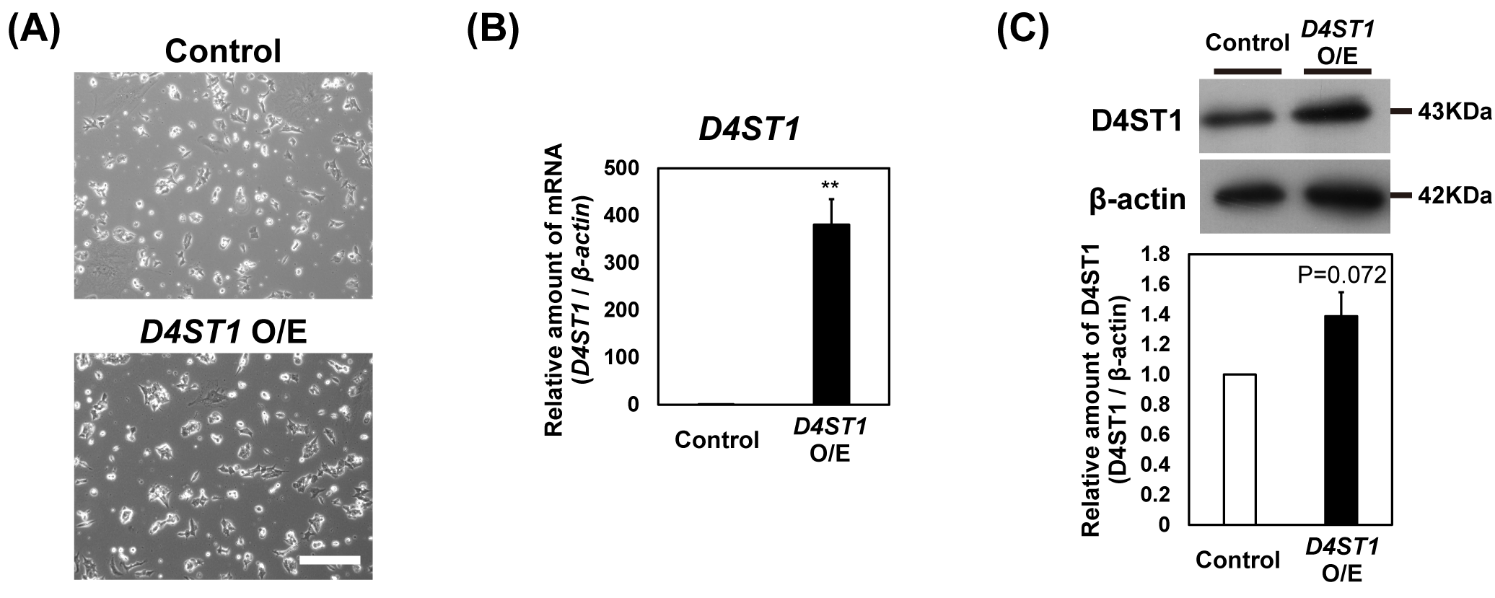


**Supplemental Figure 4. Observation of *D4ST1* O/E mESCs.**

**(A)** Morphological observation of *D4ST1* O/E mESCs at TF day2. Scale bar: 200 µm.

**(B)** Real-time PCR analysis of *D4ST1* in *D4ST1* O/E mESCs at TF day2. The amount of *D4ST1* mRNAs was normalized to that of *β-actin* mRNA and is shown relative to the control (set to 1).

**(C)** Western blotting analysis of D4ST1 at TF day2. Histogram shows the mean densitometric readings of bands, which were normalized to β-actin and are shown relative to the control (set to 1). The representative bands of the loading control (β-actin) are the same as those in Figure 3C because the same samples were used for these analyses.

The values shown are means ±SD (*N*=3). Those significantly different to the control by unpaired two-tailed Student’s t-test are indicated as follows: **, *p*<0.01.

**Supplemental Figure 5.**


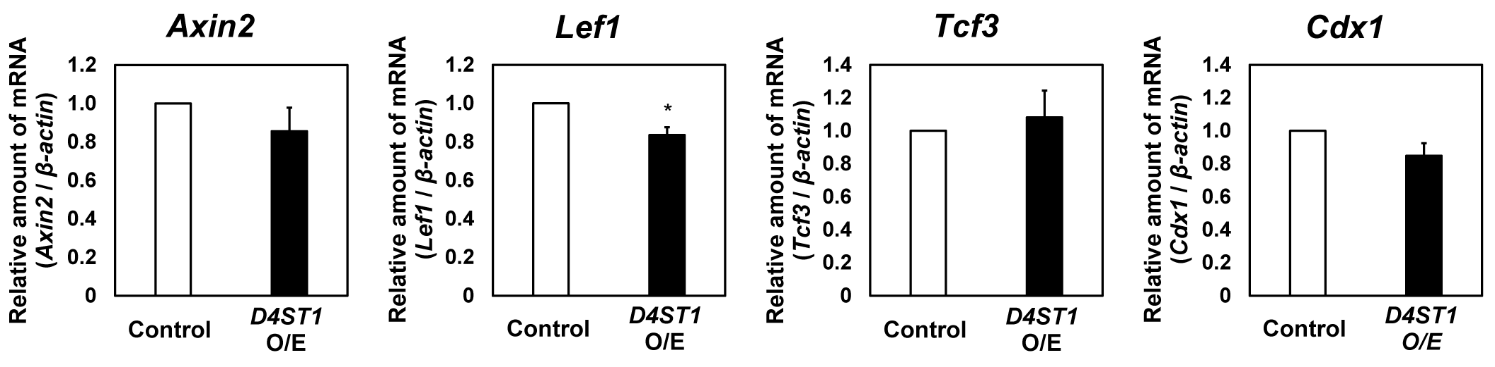


**Supplemental Figure 5. The expression of targets of Wnt signaling in *D4ST1* O/E mESCs.**

Shown is real-time PCR analysis of Wnt signaling target genes in *D4ST1* O/E mESCs at TF day2. The mRNA levels of Wnt signaling target genes (*Axin2*, *Lef1*, *Tcf3*, and *Cdx1*) were normalized to that of *β-actin* mRNA and are shown relative to the control (set to 1).

The values shown are means ±SD (*N*=3). Those significantly different to the control by unpaired two-tailed Student’s t-test are indicated as follows: *, *p*<0.05.
